# Closing the gap in HIV prevention and care for children: early insights from a model that links communities and health care facilities in Uganda

**DOI:** 10.1080/17450128.2016.1198855

**Published:** 2016-07-12

**Authors:** Doortje ‘t Hart, Merian Musinguzi, Richard Ochen, Juliet Katushabe, Joseph Rujumba

**Affiliations:** ^a^ STOP AIDS NOW!, Amsterdam, The Netherlands; ^b^ ICCO Cooperation, Central, Eastern and Southern Africa Regional Office, Kampala, Uganda; ^c^ Health Need Uganda, Soroti, Uganda; ^d^ AIDS Control Program, Ministry of Health, Kampala, Uganda; ^e^ Department of Paediatrics and Child Health, College of Health Sciences, Makerere University, Kampala, Uganda

**Keywords:** HIV prevention and care, children, community, health facility, linkages

## Abstract

Inequities in access to HIV prevention and treatment for children remain a global challenge and a black spot to effective HIV prevention and response especially in many HIV endemic countries like Uganda. In Uganda while about 51% of the adults living with HIV are on antiretrovirals, only 39% of the children aged 0–14 years accessed the needed HIV care in 2014. In this article, it is argued that much focus on health system interventions with little regard to bridging the gap between health facilities, where much of the care is provided, and the communities, where children are conceived, born and cared for, contributes to and sustains this inequality. Investments need to be made in building and implementing models that create and enhance linkages between communities and health care facilities. Success factors from the Towards an AIDS Free Generation in Uganda project model in creating these linkages are bringing all actors together in one approach, building on existing community structures and enabling community health workers to be the linking pin between communities and facilities. Only with models like this, full elimination of mother-to-child transmission and paediatric HIV care coverage (0–14 years) can be reached in Uganda and other HIV endemic countries.

## Introduction

In Africa, remarkable progress has been registered in averting new HIV infections among children with new HIV infections among children aged 0–14 years estimated at 190,000 in 2014, indicating a 47% decline since 2009 (UNAIDS, 2014). In Uganda, an estimated 69% new HIV infections were averted among children aged 0–14 years in the same period (UNAIDS, 2014; WHO, 2015), owing to the scale-up of the elimination of mother-to-child transmission of HIV (eMTCT) programme.

ART coverage for children in Africa rose to 30% in 2014, but is still lower than for adults, estimated at 41% in 2014 (WHO, 2015). In Uganda, treatment coverage for children living with HIV is still lagging at 39% compared to 51% among adults in 2014 (The Republic of Uganda, 2015). Disparities remain in the use of antiretroviral drugs (or antiretrovirals; ARVs) for eMTCT: 94% for mothers and 25% among HIV-exposed infants in 2014 (The Republic of Uganda, 2015). The current and persisting inequity stem from failure to use the available proven tools (UNAIDS, 2015).

Bridging the persistent inequities in access to HIV prevention and treatment for children in Uganda requires enrolling all pregnant women living with HIV in eMTCT, scaling up and strengthening paediatric HIV testing and care, addressing socio-economic barriers to paediatric treatment and addressing loss to follow-up for mothers living with HIV and their children. The agenda on what needs to be done is clear but the challenge remains how?

## What explains the gap in prevention and care for children amidst general improvements?

Uganda has made tremendous progress especially at health facility level, such as rolling out eMTCT to lower-level health centres, implementation of Option B+ (since 2012, all pregnant and breastfeeding women identified as HIV positive are started on lifelong ARVs irrespective of CD4 cell count) and the implementation of the Test and Treat policy whereby all children living with HIV below 15 years are initiated on lifelong ARVs regardless of CD4 count (The Republic of Uganda, 2015; UNAIDS, 2015). Despite this progress, many barriers at both health facility and community level continue to limit enrolment and retention of children in HIV care (The Republic of Uganda, 2015; UNICEF, 2015). At community level, children live in diverse household contexts, including female, grandparent and child-headed households, poverty, food insecurity, limited awareness on paediatric HIV, and distant health centres. The continued HIV stigma as well as gender and power relationships between parents limit children’s access to HIV counselling and testing as an entry point to treatment (Rujumba, Mbasaalaki-Mwaka, & Ndeezi, 2010). Furthermore, linkages between facility-based services and community outreach services are weak (Rujumba & Tushabe, 2015), leading to inadequacies in tracing, referral and follow-up of children exposed to and living with HIV.

## The TAFU project model: an example of building linkages between health care facilities and communities to bridge the gap in HIV prevention and care for children

The Towards an AIDS free generation in Uganda (TAFU) project (STOP AIDS NOW! and Partners, 2015), implemented in five rural Ugandan districts (Serere, Moroto, Napak, Mubende and Mityana), provides an example of how to bridge the HIV prevention and care gap for children in Uganda by creating linkages between communities, where children are born and cared for, and health facilities where much of the care is provided. The TAFU project partners (five Ugandan and two Dutch NGOs) collaborate with the Ugandan Ministry of Health (AIDS Control Programme) and the District Health Service departments and builds on the experience and expertise of existing HIV Implementing Partners in the five districts. The two-year project that started in 2015 aims to eliminate new HIV infections among infants and ensure all HIV-positive children in target districts are on treatment. The project addresses the socio-cultural and economic barriers households face in accessing HIV prevention and care for children, raises awareness in the community specifically on paediatric HIV, mobilises support for the children and their families, and improves coordination between communities and health care facilities to further improve identification, tracing, referral and follow-up of children exposed to or living with HIV.

## Creating linkages between communities and health care facilities is key; early insights from the TAFU project

Success factors from the TAFU project in creating linkages between communities and health care facilities are bringing all actors together in one approach, building on existing efforts of NGOs in the communities and on existing community structures (Gulaid & Kiragu, 2012) and enabling community health workers (CHWs) to be the linking pin between communities and facilities.

The lowest level of the health system in Uganda is the Village Health Teams (VHTs). These are CHWs that go into the communities to do outreach activities, make referrals and follow-up. In reaching out to families that are not yet enrolled in eMTCT and paediatric HIV care, addressing stigma and knowledge gaps on paediatric HIV at household and community levels, CHWs can play a crucial role. In the TAFU project, the original intention was to work with VHTs, but realisation has grown that more groups can act as CHWs: expert clients, mentor mothers and retired health workers. In all cases, it is vital to equip the CHWs with the right knowledge and means. In this regard, all CHWs were trained in paediatric HIV guidelines and provided with reference materials to use during health education and family-based counselling. The trained CHWs are attached to health facilities for supervision, reporting and engagement in facility-based care activities like distribution of drugs, health education and adherence counselling. Because CHWs work in and are from the communities, they are able to trace lost-to-follow-up clients and facilitate their re-engagement to care, and because CHWs work at health facilities, they are able to follow-up and provide support to women and children in communities as noted in other settings (Kim et al., 2012). During the first 6 months of the TAFU project, 358 women and 230 children living with HIV have been linked and retained in HIV care.

The project works with community resource persons including local religious and opinion leaders in community mobilisation and sensitisation on paediatric HIV prevention and care. Consistent with our early observations from TAFU, use of community resource persons has been associated with increased follow-up of HIV-infected women and early infant HIV diagnosis in other Ugandan settings (Namukwaya et al., 2015) and in Malawi (Kim et al., 2012). Recognising the potential role that schools and teachers can play in reaching out to children and their caretakers, the project is now working with primary school teachers, school management and children support groups in Serere district to stop stigma in schools for children living with HIV. Schools are also used for additional identification of children and families in need of support.

Linking up women living with HIV to self-help groups or village saving and loan associations (VSLA) has potential to increase uptake and retention of women and children living with HIV in care (hIarlaithe, Grede, de Pee, & Bloem, 2014). Within the first 6 months of operation, the TAFU project has formed and trained 32 VSLA groups with a total membership of 609 caregivers to assist families meet the financial, nutritional and psychosocial needs of children living with HIV. These groups also provide an entry point for CHWs to share information on paediatric HIV and identify children exposed to or living with HIV.

Persistent stigma, poverty and health system gaps such as stock out of HIV test kits and occasionally ARVs, few and overburdened health workers are key challenges encountered in project implementation as noted in other studies (Rujumba et al., 2010; Rujumba, Tumwine, Tylleskär, Neema, & Heggenhougen, 2012). The need to address motivation of CHWs and limited project coverage require attention for better results. Health system challenges are shared with relevant stakeholders for action while stigma is being addressed through continuous community education and counselling.

The main limitation is that our description is based on early insights from project implementation and monitoring. However, sharing these insights is important to inform other actors implementing or considering similar interventions. A participatory mixed-methods evaluation is planned at the end of the project and will provide evidence on project performance and more lessons for likely programme scale-up.

## Conclusions

In this article, it is argued that much focus on health system interventions with little regard to creating linkages between health facilities and communities contributes to and sustains the inequalities in HIV prevention and care for children in Uganda. Based on the lessons learnt so far in the TAFU project in Uganda, (1) bringing together actors working at community, health facility and national levels in one programme; (2) building on and strengthening existing structures in the community; (3) enabling CHWs to be the linking pin between communities and health facilities; (4) working with people from the community rather than outsiders and (5) constantly adapting the interventions to the context are critical factors and hold the potential for closing the gap in paediatric HIV prevention and treatment. Thus, investing in community and facility linkage models such as TAFU should be prioritised.

## References

[CIT0001] Gulaid L. A., Kiragu K. (2012). Lessons learnt from promising practices in community engagement for the elimination of new HIV infections in children by 2015 and keeping their mothers alive: Summary of a desk review. *Journal of the International AIDS Society*.

[CIT0002] hIarlaithe M. O., Grede N., de Pee S., Bloem M. (2014). Economic and social factors are some of the most common barriers preventing women from accessing maternal and newborn child health (MNCH) and prevention of mother-to-child transmission (PMTCT) services: A literature review. *AIDS and Behavior*.

[CIT0003] Kim M. H., Ahmed S., Buck W. C., Preidis G. A., Hosseinipour M. C., Bhalakia A., Kline M. W. (2012). The Tingathe programme: A pilot intervention using community health workers to create a continuum of care in the prevention of mother to child transmission of HIV (PMTCT) cascade of services in Malawi. *Journal of the International AIDS Society*.

[CIT0004] Namukwaya Z., Barlow-Mosha L., Mudiope P., Kekitiinwa A., Matovu J. N., Musingye E., Musoke P. M. (2015). Use of peers, community lay persons and Village Health Team (VHT) members improves six-week postnatal clinic (PNC) follow-up and Early Infant HIV Diagnosis (EID) in urban and rural health units in Uganda: A one-year implementation study. *BMC Health Services Research*.

[CIT0005] Rujumba J., Mbasaalaki-Mwaka C. L., Ndeezi G. (2010). Challenges faced by health workers in providing counselling services to HIV-positive children in Uganda: A descriptive study. *Journal of the International AIDS Society*.

[CIT0006] Rujumba J., Tumwine J. K., Tylleskär T., Neema S., Heggenhougen H. K. (2012). Listening to health workers: Lessons from Eastern Uganda for strengthening the programme for the prevention of mother-to-child transmission of HIV. *BMC Health Services Research*.

[CIT0007] Rujumba J., Tushabe A. (2015). *Towards an AIDS Free Generation in Uganda (TAFU) Program: A baseline survey conducted in Serere, Moroto,Napak, Mubende and Mityana Districts*.

[CIT0008] STOP AIDS NOW! and Partners (2015). *Towards an AIDS free generation in Uganda (TAFU). A programme jointly implemented by: STOP AIDS NOW! (in the Netherlands), ICCO Cooperation (Uganda office) and 5 local NGOs: Community Health Alliance Uganda (CHAU); The National Forum of People Living with HIV/AIDS networks in Uganda (Nafophanu); Health Need Uganda (HNU); Pentecostal Assemblies of God – Karamoja Integrated Development Programme (PAG-KIDEP); Deliverance Church Uganda – The AIDS Intervention Programme (DCU-TAIP). In close cooperation with the Ministry of Health in Uganda, AIDS Control Program, paediatric HIV unit*.

[CIT0009] The Republic of Uganda (2015). *HIV and AIDS Uganda country progress report 2014*.

[CIT0010] UNAIDS (2014). *Snapshort of Africa: NewHIV infections among children 2009–2014*.

[CIT0011] UNAIDS (2015). *Treatment 2015*.

[CIT0012] UNICEF (2015). *Situation analysis of children in Uganda*.

[CIT0013] WHO (2015). *Progress report: Global health sector response to HIV, 2000-2015- focus on innovations in Africa*.

